# Introducing a Clustering Step in a Consensus Approach for the Scoring of Protein-Protein Docking Models

**DOI:** 10.1371/journal.pone.0166460

**Published:** 2016-11-15

**Authors:** Edrisse Chermak, Renato De Donato, Marc F. Lensink, Andrea Petta, Luigi Serra, Vittorio Scarano, Luigi Cavallo, Romina Oliva

**Affiliations:** 1 Kaust Catalysis Center, King Abdullah University of Science and Technology, Thuwal, 23955-6900, Saudi Arabia; 2 Dipartimento di Informatica ed Applicazioni, University of Salerno, Via Giovanni Paolo II, 132, 84084, Fisciano (SA), Italy; 3 University Lille, CNRS UMR8576 UGSF, F-59000, Lille, France; 4 Department of Sciences and Technologies, University “Parthenope” of Naples, Centro Direzionale Isola C4 80143, Naples, Italy; University of Michigan, UNITED STATES

## Abstract

Correctly scoring protein-protein docking models to single out native-like ones is an open challenge. It is also an object of assessment in CAPRI (Critical Assessment of PRedicted Interactions), the community-wide blind docking experiment. We introduced in the field the first pure consensus method, CONSRANK, which ranks models based on their ability to match the most conserved contacts in the ensemble they belong to. In CAPRI, scorers are asked to evaluate a set of available models and select the top ten ones, based on their own scoring approach. Scorers’ performance is ranked based on the number of targets/interfaces for which they could provide at least one correct solution. In such terms, blind testing in CAPRI Round 30 (a joint prediction round with CASP11) has shown that critical cases for CONSRANK are represented by targets showing multiple interfaces or for which only a very small number of correct solutions are available. To address these challenging cases, CONSRANK has now been modified to include a contact-based clustering of the models as a preliminary step of the scoring process. We used an agglomerative hierarchical clustering based on the number of common inter-residue contacts within the models. Two criteria, with different thresholds, were explored in the cluster generation, setting either the number of common contacts or of total clusters. For each clustering approach, after selecting the top (most populated) ten clusters, CONSRANK was run on these clusters and the top-ranked model for each cluster was selected, in the limit of 10 models per target. We have applied our modified scoring approach, Clust-CONSRANK, to SCORE_SET, a set of CAPRI scoring models made recently available by CAPRI assessors, and to the subset of homodimeric targets in CAPRI Round 30 for which CONSRANK failed to include a correct solution within the ten selected models. Results show that, for the challenging cases, the clustering step typically enriches the ten top ranked models in native-like solutions. The best performing clustering approaches we tested indeed lead to more than double the number of cases for which at least one correct solution can be included within the top ten ranked models.

## Introduction

The thousands of proteins expressed in cells perform most of their functions through interactions with other proteins [1,2]. Understanding protein-protein interactions and characterizing them on a structural basis is thus a crucial step in the investigation of many biological processes [3,4]. However, experimental structures of protein-protein complexes are still under-represented [5]. Many more protein complex structures could in principle be predicted by computational approaches, specifically by macromolecular docking, However, reliably predicting the three-dimensional structure of protein-protein complexes is still challenging, with one of the critical steps being the scoring, i.e. the ability to discriminate between correct and incorrect solutions within a pool of models [6–8].

The CAPRI (Critical Assessment of PRedicted Interactions) experiment [9,10] organizes blind docking challenges and has been catalyzing the development of computational protein docking for over a decade [11,12]. Since 2006, a scoring session has been included in the experiment [10,13], allowing the assessment of scoring functions irrespective of the used docking protocols. Briefly, Dockers may submit a set of 100 models each; the ensemble of models is then anonymized and made available to Scorers. Scorers are invited to re-rank the models using their preferred scoring function and to resubmit their own top 10 models. Success is measured on the number of targets or interfaces for which at least one native-like model—a model of at least acceptable quality—was submitted.

Traditionally, scoring functions for protein-protein docking poses are energy and/or knowledge based, therefore they calculate a score for each model *per se* [14–16]. We introduced in the field CONSRANK, the first pure consensus method [17]. CONSRANK, also available as a web server [18], ranks models based on their ability to match the most conserved (or frequent) inter-residue contacts in the ensemble they belong to, thus being the first scoring algorithm relying on the contacts in the docking decoys ensemble. However, inter-residue contacts observed in docking poses of protein-protein complexes have been previously used for different scopes. CAPRI assessors have been using contacts (specifically the fraction of them which are native, i.e. common to the corresponding experimental structure) as one of the criteria for assessing the docking predictions correctness, since the first experiment edition [11]. We have proposed to use contacts as a tool to analyse and compare docking model ensembles [19] (more recently, we extended the approach to other conformational ensembles of protein-protein complexes [20–22]); while Bonvin and co-workers introduced them for the models clustering [23]. In particular, they proposed the use of the fraction of common contacts within models as a similarity description to base their clustering on. As the native structure of a complex is not expected to be an isolated event in the energy landscape, docking experiments indeed often incorporate a clustering step. In this context, Bonvin et al. inspiringly showed that a contact-based clustering can greatly reduce the computation time while generating clusters of similar quality with the state-of-the art RMSD-based methods [23].

CONSRANK was recently blindly tested in the CASP11/CAPRI30 joint prediction round, where the prediction of protein complexes was assessed for 25 targets (T68 to T94), consisting of mostly homodimers, a few homotetramers and two heterodimers. CONSRANK, together with the Bates’ group, featured the highest number of correct models submitted (136 and 131, respectively), having on average 9 over 10 correct models for the successful targets; it also achieved the overall best result for 6 targets (T75, T84, T87, T89, T90 and T91) and could identify native-like solutions for 14 targets, for 11 of them of high or medium quality [12]. However, in spite of the high success rate for selected targets, CONSRANK was ranked 9^th^ overall, due to failure for the more complicated cases. In comparison, the Bonvin’s group—the first-ranked scorer group—listed 18 successful targets, 14 of which with high or medium quality solutions. Apart from the three targets (T68, T77, T88) for which no correct solution was identified in the ensemble of the experiment, CONSRANK also failed on five other targets, for which other Scorers could identify few correct solutions. Of these targets, two (T70 and T71) were predicted homotetramers, which our contact-based approach at present cannot handle. Three (T72, T79 and T86) were predicted dimers. We would like to stress that the oligomeric state assignment for some of these targets is ambiguous and mostly unconfirmed; for instance, T70, assumed tetrameric at the time of the experiment, was finally listed as a dimer in the corresponding PDB entry (PDB ID: 4PWU).

In short, critical cases for CONSRANK feature a small or very small number of correct solutions. They typically have multiple putative interfaces, with uncertainties as to their physiological relevance. A small fraction of correct solutions in the models ensemble and the presence of multiple interfaces have in fact already been associated to a decreased CONSRANK performance, when applied to other scoring benchmarks, as they represent intrinsic limitations of a consensus approach [17,24].

To address these challenging cases, CONSRANK has now been modified to include a contact-based clustering of the models as a preliminary step of the scoring process. The clustering method we used substantially differs from the contact-based one proposed by Bonvin and colleagues [23] as it: i) uses the absolute number of different contacts as a distance measure between pairs of models (resulting in a symmetric rather than an asymmetric similarity matrix), and ii) relies on a hierarchical clustering algorithm. This novel approach, Clust-CONSRANK, has been tested on the above-mentioned three “critical targets” of CAPRI Round 30, all corresponding to putative homodimers, but also on the set of CAPRI scoring models, SCORE_SET, made recently available by the CAPRI assessors [25]. The SCORE_SET targets span a wide time period, going from T29, included in ROUND 13, to T54, in ROUND 26, and are involved in a variety of biological functions [13,26]. The difficulty of the various targets is also very variable, and ranges from relatively easy, where the coordinates of at least one component were given to predictors in the bound state (such as T29), or high-quality templates existed (T47), to intermediate, where coordinates of both components were given in their unbound state (such as T30, T32, T35, T37, T39, T41, T50, T53, T54), and to difficult, where one or both components were to be modeled by homology (for example T37 and T46). Besides these, T40 has one component simultaneously bound to two copies of the second protein, forming two distinct association modes, and designed proteins are also represented (T50).

To score these models, different clustering approaches have been explored and relative results are discussed comparatively. Scoring results have also been compared to those obtained by the original CONSRANK algorithm. Obtained results clearly show that the clustering step allows enhancing the number of targets/interfaces with at least one correct solution identified.

## Materials and Methods

### Dataset

Thirteen decoy sets for old CAPRI scoring models corresponding to fourteen interfaces and relative CAPRI classification in incorrect, acceptable, medium and high-quality models, were downloaded from the SCORE_SET site: http://cb.iri.univ-lille1.fr/Users/lensink/Score_set [25]. 3D models for the three CAPRI Round 30 targets (T72, T79 and T86) were also analysed. A classification in incorrect, acceptable, medium and high-quality models according to the CAPRI criteria [13] was obtained for all their six interfaces. A total of 20721 3D models were analysed.

### Models renumbering

All the models for a given target/interface were modified to be consistently renumbered, i.e. to have corresponding amino acids featuring the same number and chain identifier, which is a fundamental prerequisite for subsequent analyses. To this aim, we used our in-house renumbering tool, also available online at https://www.molnac.unisa.it/BioTools/consrank/renumbering/renumbering.html [18], which first extracts the FASTA sequences from the PDB files, then uses BLASTclust (ftp://ftp.ncbi.nih.gov/blast/documents/blastclust.html) to clusterize them and aligns sequences within each cluster with ClustalW (http://www.ebi.ac.uk/Tools/msa/clustalw2/), finally rewrites the PDB files to make the numbering consistent. The sequence identity and coverage used in BLASTclust were 70% and 0.9, respectively.

### Models scoring and selection

For the original CONSRANK function, the whole set of models per each target was submitted to the CONSRANK code [17] and the 10 top ranked models were selected as predicted positive. In the clustering approaches (see below), after selecting the 10 top (most populated) clusters, the CONSRANK code was run on models belonging to each cluster and the model top ranked by CONSRANK for each cluster, for a total of 10 models, was selected. All the complex 3D representations were prepared with PyMol [27]. Contact maps of the X-ray interfaces were obtained by COCOMAPS [28]. Consensus maps for the model ensembles were obtained by the CONSRANK server [18].

### Clustering

Two residues are considered in contact if they have any pair of heavy atoms within a distance of 5 Å. Then, a Hamming distance between the models is calculated based on the above defined contacts. For instance, a Hamming distance of 20 between two models means that they differ by 20 inter-residue contacts. Therefore, the absolute number of different contacts is used here as a distance measure between pairs of models, instead of the fraction of common contacts normalized over the number of contacts of either model, used as a similarity measure by Bonvin and colleagues [23]. Python libraries ScyPy [29] and fastcluster [30] have been used in the following steps. Based on the above calculated metric, a distance n(n-1)/2 sized vector, where *n* is the number of models, has been obtained by the cluster.pdist function (SciPy library). Elements of this vector represent the Hamming distances between all pairs of models.

At this point, starting from the above distance vector, we generated linkage matrixes by the linkage function (fastcluster library), based on two methods: single and complete, both having a O(n^2^) time complexity. The ‘single’ method assigns:

d(u,v)=min(dist(u[i],v[j]))

for all points *i* in cluster *u* and *j* in cluster *v*. It is also known as the Nearest Point Algorithm. The ‘complete’ method instead assigns:

d(u,v)=max(dist(u[i],v[j]))

for all points *i* in cluster *u* and *j* in cluster *v*. It is also known as the Farthest Point Algorithm. Finally, we performed an agglomerative (bottom-up) hierarchical clustering, by the cluster.hierarchy.fcluster function (SciPy library), differently from Bonvin and colleagues, who used a version of the disjoint non-hierarchical Taylor-Butina algorithm adapted to handle asymmetric matrices [23]. Two criterions were used in the clusters generation: Distance and Maxclust. When the Maxclust criterion is used, the maximum number of flat clusters, *t*, is set. Maxclust finds automatically a distance value so that no more than *t* flat clusters are formed.

For the Distance criterion, different thresholds were tested both with the single and complete methods. Results are reported only for the thresholds shown to increase the number of targets/interfaces with at least one correct solution within the top 10 selected. These thresholds were 25 and 30 for the single method (meaning that the closest pair of elements belonging to different clusters must be farther than a distance of 25 and 30 respectively), and 40, 50, 60 and 80 for the complete method (meaning that the farthest pair of elements belonging to different clusters must be farther than 40, 50, 60 and 80, respectively).

The Maxclust approach was based on the complete method. Also in this case, different thresholds were tested. As we got promising results with a fixed number of 200 clusters per target/interface, that corresponds roughly to 1/5 to 1/10 of the total models available per target/interface, to make the approach independent of the ensemble size, we also explored the following *t* thresholds: i) 1/5 and ii) 1/10 of the total number of models per target.

We also tested the Maxclust approach, with the same thresholds set above, on the SCORE_SET targets, using the ligand RMSD values as distance measures. The ligand RMSD for each pair of models is the root mean square deviation calculated on the backbone atoms of the ligand (i.e. the smaller interactor) in the two models, after the receptor (i.e. the larger interactor) backbones have been best superimposed (Tables A-B in S1 File).

The output of the clustering procedure is a list, where every row represents a cluster and contains indices corresponding to all the 3D models included in it. Clusters are ranked based on their population. The top (most populated) 10 clusters were selected for further analyses. Our clustering algorithm was implemented in the Python programming language and is freely available upon request.

### Redundancy removal

A redundancy removal approach was also tested. In particular, after selecting the model top ranked by CONSRANK, all the models too similar to it (redundant), i.e. within a given distance threshold, were discarded and the top remaining prediction was selected. The process was carried on until ten models were selected. As a distance measure between models, we used again the number of different inter-residue contacts. All the distance thresholds explored in the clustering step, spanning the range 25 to 80, were tested (Tables C-D in S1 File).

## Results and Discussion

To test the performance of Clust-CONSRANK, we applied it to two sets of models used in previous CAPRI scoring experiments, containing at least one correct solution to be possibly singled out. The first set is made of 13 targets (and 14 interfaces) from SCORE_SET, a CAPRI scoring benchmark publicly available at http://cb.iri.univ-lille1.fr/Users/lensink/Score_set [25]; targets T36 and T39 were discarded because they had no acceptable solution. The second set consists of the 3 dimeric targets (and 6 interfaces) in the recent CAPRI round 30, for which our pure consensus scoring function, CONSRANK, failed to identify any correct solution. Therefore, we considered here a total of 16 targets and 20 interfaces. The average number of models for target/interface is ≈ 1300, while the percentage of native-like solutions ranges between 0.15%, for T30, and 57%, for T47 (see Table 1).

**Table 1:** Scoring results for the analysed targets/interfaces.

Target.Interface	# models	NL	%NL	H	M	A	I	R	CONSRANK	Clust-CONSRANK								
										S^25^	S^30^	C^40^	C^50^	C^60^	C^80^	MC^200^	MC^/5^	MC^/10^
**T29**	2083	144	6.9	2	72	70	1629	310	10/9*	2/2*	2/2*	4/3*	4/2*	4/2*	3/2*	2/1*	2/1*	2/1*
**T30**	1343	2	0.15	0	0	2	1104	237	0/0*	0/0*	0/0*	0/0*	0/0*	0/0*	0/0*	0/0*	0/0*	0/0*
**T32**	599	15	2.5	0	3	12	557	27	0/0*	0/0*	2/0*	0/0*	0/0*	1/0*	1/0*	2/0*	2/0*	1/0
**T35**	499	2	0.40	0	0	2	465	32	0/0*	0/0*	0/0*	0/0*	0/0*	0/0*	0/0*	0/0*	0/0*	0/0*
**T37**	1500	78	5.2	8	35	35	1060	362	9/9*	0/0*	0/0*	3/2*	1/1*	0/0*	0/0*	1/1*	1/1*	2/1*
**T39**	1400	4	0.29	0	3	1	1257	139	0/0*	0/0*	0/0*	0/0*	0/0*	0/0*	0/0*	0/0*	0/0*	1/1*
**T40.CA**	2180	354	16	90	141	123	1531	295	10/10*	2/2*	1/1*	5/4*	6/4*	7/5*	6/4*	1/1*	4/4*	2/2*
**T40.CB**	2180	134	6.2	86	22	26	1751	295	0/0*	1/1*	1/1*	2/2*	1/1*	1/1*	1/1*	1/1*	1/1*	1/1*
**T41**	1200	299	25	2	99	198	730	171	10/5*	1/0*	1/0*	3/1*	5/2*	5/2*	4/2*	4/1*	5/2*	3/1*
**T46**	1699	24	1.4	0	0	24	1297	378	0/0*	0/0*	1/0*	0/0*	0/0*	1/0*	0/0*	1/0*	0/0*	0/0*
**T47**	1051	600	57	278	301	21	388	63	10/10*	1/1*	1/1*	8/8*	6/6*	4/4*	1/1*	6/6*	8/8*	2/2*
**T50**	1451	124	8.6	0	35	89	1141	184	0/0*	2/0*	2/1*	0/0*	1/0*	1/1*	2/0*	3/1*	2/0*	2/2*
**T53**	1400	101	7.2	0	9	92	1090	209	3/0*	1/0*	1/0*	0/0*	1/0*	2/0*	1/0*	1/0*	1/0*	1/0*
**T54**	1400	19	1.4	0	1	18	1195	185	0/0*	0/0*	0/0*	1/0*	0/0*	0/0*	0/0*	1/0*	0/0*	1/0*
**T72.1**	914	6	0.66	0	0	6	659	249	0/0*	0/0*	0/0*	0/0*	0/0*	0/0*	0/0*	0/0*	0/0*	0/0*
**T79.1**	999	20	2.0	0	7	13	701	278	0/0*	0/0*	0/0*	0/0*	0/0*	0/0*	0/0*	0/0*	0/0*	0/0*
**T79.2**	999	63	6.3	0	7	56	658	278	0/0*	2/0*	2/0*	3/0*	3/0*	1/0*	2/0*	2/0*	2/0*	1/0*
**T79.3**	999	2	0.20	0	0	2	719	278	0/0*	0/0*	0/0*	0/0*	0/0*	0/0*	0/0*	0/0*	0/0*	0/0*
**T86.1**	1010	30	3.0	0	5	25	942	38	0/0*	1/0*	1/0*	1/0*	1/0*	1/0*	0/0*	0/0*	0/0*	1/0*
**T86.2**	1010	25	2.5	0	8	17	947	38	0/0*	1/0*	1/0*	2/1*	1/0*	2/1*	1/0*	1/0*	1/0*	1/0*

Columns 1–9: features of analysed targets: H, M, A, I and R indicate the high, medium-quality, acceptable, incorrect and removed models. NL stays for native-like, that is the sum of H, M and A models. Columns 10–19: results of the scoring with original CONSRANK scoring algorithm and with different combined Consrank-clustering approaches. In each column the total number of NL/H+M* models per target/interface is reported. “S” stays for Single, “C” for Complete, with numbers indicating the corresponding thresholds used in the clustering; MC^200^, MC^/5^ and MC^/10^” indicate results of a clustering approach with a maximum number of clusters fixed to 200 and to 1/5 and 1/10 of the total number of models per target, respectively (see Methods) Positive results are highlighted in cyan.

**Fig 1:**
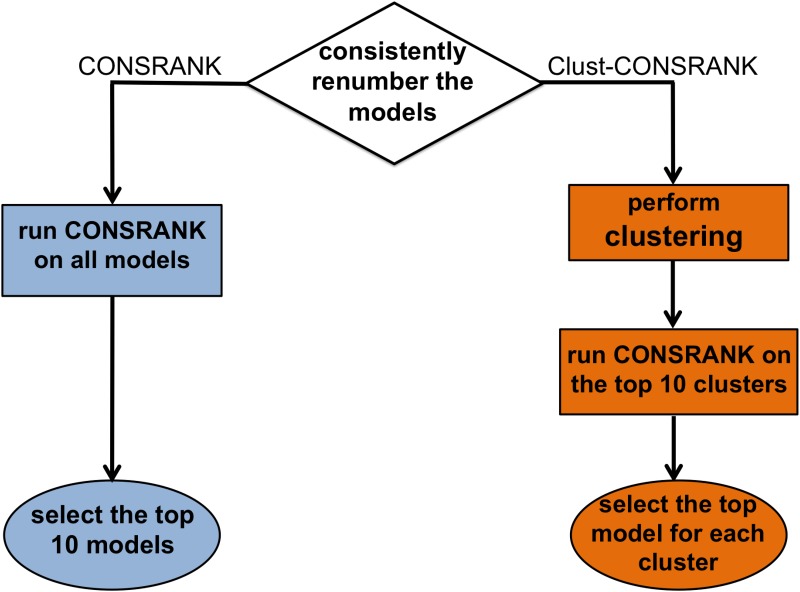
Schematic representation of the CONSRANK and Clust-CONSRANK workflow.

A scheme of the workflow for the CONSRANK and Clust-CONSRANK approaches is given in Fig 1. For each ensemble of models, we first edited the PDB files to have them consistently renumbered, i.e. with corresponding residues having the same number and chain identifier. As a second step, we simply ran CONSRANK on them and selected as predicted positive the top ten ranked solutions. For the Clust-CONSRANK approach, we first applied to the renumbered models different clustering procedures with various thresholds. The top ten (most populated) clusters from each approach were selected and CONSRANK was run on models belonging to these clusters. The CONSRANK top ranked model for each cluster (for a total of 10 models) was selected as predicted positive. All the clustering approaches were hierarchical and we used as a measure of the distance between different models the number of different inter-residue contacts between them. In two clustering approaches, “Single” (abbreviated in the following as S^N^, where N is the threshold) and “Complete” (abbreviated as C^N^, where N is the threshold), a threshold was set on the distance between the models, respectively the minimum distance between the closest points and the maximum distance between the farthest points in two different clusters. In a third clustering approach, MaxClust (abbreviated as MC^N^, where N is the threshold or MC^/M^, when the threshold is given by the total number of models divided by M), a threshold was instead set on the maximum number of allowed clusters.

### Scoring results with and without the clustering step

Results of original CONSRANK and modified clust-CONSRANK scoring functions are reported in Tables 1 and 2. We are particularly interested in testing the ability of the clustering step to enhance the number of targets/interfaces for which at least one correct solution is included in the top 10 ranked models, as compared to CONSRANK.

**Table 2:** Number of interfaces for which at least one acceptable/high-medium quality (*) solution has been selected by each scoring approach.

Method	Total Interfaces with ≥ 1 NL/HM
**CONSRANK**	6/5*
**Clust-CONSRANK**	
**S**^**25**^	10/4*
**S**^**30**^	12/5*
**C**^**40**^	10/7*
**C**^**50**^	11/6*
**C**^**60**^	12/7*
**C**^**80**^	10/5*
**MC**^**200**^	13/7*
**MC**^**/5**^	11/6*
**MC**^**/10**^	14/8*

Not surprisingly, with the only exception of T50, CONSRANK could identify correct solutions for all the targets featuring more than 5% correct solutions and a single interface. For these 6 targets, the average number of correct solutions identified was as high as 8.7. It failed, however, on targets with less than 2.5% correct solutions in the set or on those featuring more than one interface. In terms of CAPRI assessment, this means having six over 20 interfaces with at least one correct solution identified, 5 of them with models of medium quality.

An inspection of Tables 1 and 2 clearly shows that all the explored clustering approaches, with the appropriate thresholds, lead to an increase in the number of targets or interfaces for which we could identify at least one correct solution, as compared to the original CONSRANK approach. The best results for the “Single” approach were achieved with a distance threshold of 30. The S^30^ approach indeed allowed doubling the number of interfaces (from 6 to 12) for which at least one correct solution was identified, as compared to CONSRANK. For the “Complete” approach, the best results were achieved with a threshold of 80. With the C^80^ approach it was possible to both double the number of interfaces with at least one correct solution, and to identify at least one medium or high quality solution for two additional interfaces, as compared to CONSRANK.

The overall best results were however achieved with the third approach, MaxClust, i.e. by setting the number of clusters themselves instead of the different contacts. We explored different cluster numbers, finding positive results with the number set to 200. The MC^200^ approach indeed allowed identifying correct solutions for a total of 13 interfaces and high/medium quality solutions for 7 of them. As 200 clusters corresponds roughly to 10 to 20% of the total models available per target/interface, to make the approach independent of the ensemble size we also explored the following thresholds: 1/5 (20%; MC^/5^) and 1/10 (10%; MC^/10^) of the total number of models per target. In particular, the MC^/10^ approach further improved the MC^200^ performance and allowed identifying correct solutions for a total of 14 interfaces and high/medium quality solutions for 8 of them. With this approach, it was possible to identify correct solutions for all the targets with a percentage of correct solutions above 2.0% and even for two targets, T39 and T54, featuring only 0.29 and 1.4% correct solutions, respectively. It is worth mentioning that for these two targets no scorer in CAPRI could single out any correct solution from the same model ensembles at the time [13,26]. It is also worth pointing out that all the six cases where the MC^/10^ approach failed are quite challenging ones. In the corresponding CAPRI scoring experiments, for three of them—T30, T35 and T79.3–0, 1 and 2 correct solutions overall were identified by the scorer groups. For the three remaining cases, T46, T72.1 and T79.1 only a handful of scorers could identify in total a dozen correct models (in the most successful case, T46, 8 scorers identified collectively 15 correct models).

For the sake of comparison, we also tested the most successful clustering approaches, MC^200^, MC^/5^ and MC^/10^, on the SCORE_SET targets, by using as a distance measure the ligand RMSD instead of the inter-residue contacts. Results, reported in Tables A-B in S1 File, show that the number of targets/interfaces for which at least one correct solution could be identified is the same for the RMSD and the contact-based clustering approaches (with only the RMSD-based MC^200^ having one successful target less compared to the contact-based one).

### Redundancy removal

We also investigated whether, analogously to the clustering step, a simple redundancy removal strategy could improve the CONSRANK performance. Starting from the CONSRANK scoring, we thus considered redundant and removed all predictions too similar to the models already selected, i.e. within a given distance threshold. The distance between a pair of models was defined as the number of different inter-residue contacts they feature (see Methods). The whole range of distance thresholds explored in the clustering step, from 25 to 80, was tested. Results of this analysis are reported in Tables C-D in S1 File and show that the redundancy removal only slightly increases (from 6 to 8) the number of interfaces for which at least one correct solution is identified, as compared to CONSRANK, while leaving unaffected the number of interfaces with at least one medium/high solution identified (depending on the distance threshold for redundancy it ranges from 4 to 6, versus the 5 identified by CONSRANK).

### Details on two scoring cases

In the following, details are given on two scoring cases, T50 and T86, where the clustering, and in particular the MC^/10^ approach, significantly improved the scoring results as compared to CONSRANK. While discussing these cases, we will make use of contact maps and “consensus maps”. Therefore, it is worth reminding here that an intermolecular contact map is a contact map where a black dot is present at the cross-over of two residues on two different molecules, having any pair of heavy atoms closer than a cut-off distance. Consensus maps, that we introduced and used for analyzing and visualizing the interface conservation in structure ensembles of protein complexes [19–22,31–33], are intermolecular contact maps where inter-residue contacts are reported on a grey scale. The darker the dot, the more conserved the contact in the ensemble of analysed models/structures.

#### T50

The T50 target is a SCORE_SET target corresponding to the *de novo* designed binding protein HB36.3 in complex with influenza virus hemagglutinin (HA), cleaved into its two subunits, HA1, a large globular domain, and HA2, a long, helical domain anchoring the protein to the membrane (PDB ID: 3R2X, [34]). The HM36.3 protein was successfully designed to bind a conserved surface patch on the stem of HA (HA2 subunit).

**Fig 2:**
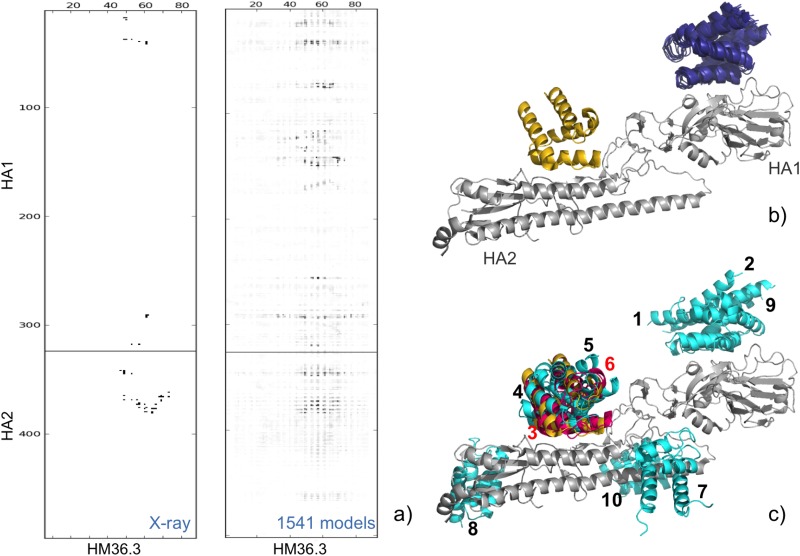
T50 scoring. (**a)** X-ray structure contact map obtained by COCOMAPS [28] (left) and consensus map from the 1451 available models (right). (**b, c)** 3D representation of the T50 target experimental structure and of selected models by CONSRANK (b) and by Clust-CONSRANK—MC^/10^ (c). X-ray receptor and ligand are colored silver and gold, respectively. Ligands of models selected by CONSRANK are colored deep blue, while incorrect and correct solutions selected by MC^/10^ are colored light blue and hot pink, respectively.

As we have previously discussed [17], having many models pointing to the same false consensus is not the most probable event, as incorrect contacts are usually wrong in a different way, thus giving destructive interference and indeed we rarely observed this to happen [17,24]. However, this is the case for this target, where hundreds of models point to a false interface, with HB36.3 binding the HA1 subunit of influenza hamagglutinin (particularly regions around residues 20, 89 and 120–160). This is quite clear by an inspection of the crystal structure contact map compared to the consensus map obtained from the 1451 scoring models, shown in Fig 2. This is of course a worst case scenario for a consensus approach and helps to explain why this was the only target where CONSRANK failed to include any correct solution within the top 10 ranked models, even though the scoring set included a significant fraction of correct solutions (8.6%). Models selected by CONSRANK, shown in Fig 2a, indeed point to the wrong interface, as do also the selected models from the 1^st^, 2^nd^, and 9^th^ clusters from the MC^/10^ clustering approach, containing respectively 523, 168 and 25 models (collectively 716). However, models selected from other MC^/10^ clusters do explore other interfaces, with four of them correctly pointing to the stem region on the HA2 subunit, where the binding of the HB36.3 designed protein is directed, and including two medium quality models, from the 3^rd^ and 6^th^ clusters (containing 75 and 43 models, respectively).

#### T86

T86, the polyketide Cyclase from *Sinorhizobium meliloti*, was predicted to be homodimeric by both PISA and the structure authors (PDB ID: 4UI3). However, it features very small subunit interface areas, with the largest one being around 470 Å^2^. The two largest interfaces according to PISA [35] were assessed in CAPRI, named interface 1 and 2. This is one of the three dimeric CAPRI Round 30 targets for which we were unable to submit any correct solution for either interface by the classical CONSRANK approach [12].

**Fig 3:**
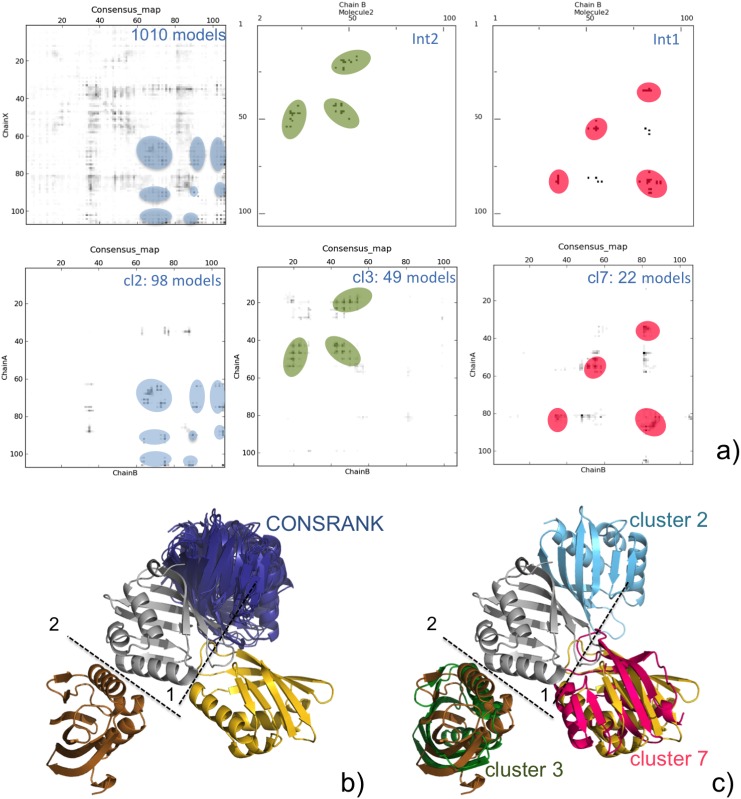
T86 scoring. **(a)** Consensus map (from the 1010 models) and contact map of the two target assessed interfaces (above) and consensus maps from the models in the 2^nd^, 3^rd^ and 7^th^ MC^/10^ clusters (below). Regions highlighted in the maps correspond to specific models/interfaces. For the color code, see below. (**b, c)** 3D representation of the T86 target experimental structure and of selected models by CONSRANK (b) and by Clust-CONSRANK—MC^/10^ (c). X-ray receptor is colored in silver, while the ligand at the interface 1 and 2 is colored in gold and copper, respectively. Ligands of models selected by CONSRANK are colored deep blue, incorrect solutions selected by MC^/10^ are colored light blue, while correct solutions according to interface 1 and 2 are colored hot pink and green, respectively.

The MC^/10^ clustering approach allows instead including within the top 10 ranked models one acceptable solution for both interfaces. More in detail, near-native solutions for interfaces 2 and 1 were selected from the 3^rd^ and 7^th^ most populated MC^/10^ clusters, respectively, while the top ranked solution of the 2^nd^ MC^/10^ cluster (containing 98 models) was the same top selected by CONSRANK over the whole ensemble of 1010 models (see Fig 3). Remarkably, only half of the models in the 3^rd^ MC^/10^ cluster (25 over 49), and only one third of the models in the 7^th^ MC^/10^ cluster (8 over 22) are correct according to the CAPRI criteria (corresponding to interface 2 and 1, respectively), and they were top ranked by CONSRANK.

We conclude that the success in identifying correct solutions is here clearly the result of a combination between i) the ability of the MC^/10^ clustering approach to create enough populated clusters that are enriched in correct solutions, and ii) the ability of CONSRANK to top rank the correct models even from ensembles of reduced size, provided that they contain a reasonable fraction of correct solutions [24]. In Fig 3, the contact maps corresponding to the target interfaces 1 and 2 are shown, in comparison with the consensus maps obtained from the ensemble of 1010 T86 models and from the models in the 2^nd^, 3^rd^ and 7^th^ MC^/10^ clusters. Corresponding regions in the maps are highlighted in the same color (also common to the shown related 3D structures). From these maps, it is clear that also incorrect models contained in clusters 3 and 7 point to the correct interface (1 and 2, respectively). This is not surprising, as it has been shown by the CAPRI assessors that about one quarter of the interfaces in models ranked as incorrect in CAPRI are actually correctly predicted (with these models contributing 70% of the correct interface predictions overall [36]). The presence in the ensembles of docking predictions of “incorrect” models featuring correct contacts is in fact most probably key to the success of the CONSRANK scoring approach, which clearly outperforms pure consensus approaches based on RMSD measures, as we have already extensively discussed [24].

## Conclusions

In an attempt to overcome the intrinsic limitations of a pure consensus approach, such as the classical CONSRANK algorithm, and to increase the number of targets for which at least one correct solution is included in the top 10 selected models, we have implemented a modified scoring algorithm, Clust-CONSRANK. In Clust-CONSRANK, CONSRANK is preceded by a contact-based clustering step. Different clustering procedures and thresholds were explored, all using a hierarchical approach. The clustering step implemented in Clust_CONSRANK uses the number of different inter-residue contacts as a measure of the distance between models. However, as we also show, similar results may be achieved by using the ligand RMSD as a measure of the models distance, with the same clustering approach.

We applied Clust-CONSRANK to an extended and diverse set of CAPRI scoring model ensembles, and found the most successful clustering method to be MC^/10^, i.e. the MaxClust approach with the number of clusters defined as 1/10 of the total number of models per target. While all the presented clustering procedures allowed increasing the number of successful cases, MC^/10^ more than doubled the number of interfaces with at least one correct solution identified (from 6 to 14), as compared to the pure CONSRANK approach, and significantly increased the number of interfaces (from 5 to 8) with at least one medium/high quality solution singled out. Remarkably, a simple redundancy removal approach cannot instead significantly improve the CONSRANK performance in such terms.

The reason for the success of the Clust-CONSRANK approach thus seems to be two-fold. First, the clustering step enhances the sampling of the conformational space and includes in the generated clusters few ones enriched in correct contacts. Then, the consensus approach of CONSRANK, when applied on clusters enriched in correct contacts, is able to top rank correct solutions as opposed to incorrect ones.

We have shown on different scoring benchmarks [17,24], in the recent CAPRI Round 30 collaboration with CASP11 [12], and in the latest CAPRI Rounds 31–35 (http://www.ebi.ac.uk/msd-srv/capri/), that our consensus scoring function, CONSRANK, is on par with the performance of state-of-the-art energy- and knowledge-based scoring functions for targets with well-defined interaction interfaces and sufficiently enriched docking ensembles. We show here that for the remaining and challenging targets, the introduction of a clustering step prior to the scoring significantly enhances the likelihood of including native-like solutions in the top 10 ranked complexes.

## Supporting Information

S1 Data Main outputs relative to all the presented analyses. (TGZ)

S1 File Tables reporting results of the RMSD-based clustering and of the redundancy removal approach. (PDF)
