# Supplementary material for: Introducing a Clustering Step in a Consensus Approach for the Scoring of Protein-Protein Docking Models
Source: PLoS One. 2016 Nov 15;11(11):e0166460. doi: 10.1371/journal.pone.0166460 (PMC5112798; doi:10.1371/journal.pone.0166460)
Supplement: S1 File — (PDF) [file pone.0166460.s002.pdf]

S1 File

for

"Introducing a Clustering Step in a Consensus Approach for the Scoring of Protein-Protein Docking Models"

Edrisse Chermak, Renato De Donato, Marc F. Lensink, Andrea Petta, Luigi Serra, Vittorio Scarano, Luigi Cavallo and Romina Oliva

Table A. Scoring results for the SCORE_SET targets/interfaces. Columns 1-9: features of analysed targets: H, M, A, I and R indicate the high, medium-quality, acceptable, incorrect and removed models. NL stays for native-like, that is the sum of H, M and A models. Columns 10-16: results of the scoring with original CONSRANK scoring algorithm and with different combined Consrank-clustering approaches, based on inter-residue contacts (columns 11-13) and ligand RMSD (columns14-16). In each column the total number of NL/H+M models per target/interface is reported. MC^{200}, $MC^{/5}$ and $MC^{/10}$ " indicate results of clustering with a maximum number of clusters fixed to 200 and to 1/5 and 1/10 of the total number of models per target, respectively. Positive results are highlighted in cyan.

Target.Interface	# models	NL	%NL	H	M	A	I	R	CONSRANK	Clust-CONSRANK			RMSDClust CONSRANK		
										MC^{200}	$MC^{/5}$	$MC^{/10}$	MC^{200}	$MC^{/5}$	$MC^{/10}$
**T29**	2083	144	6.9	2	72	70	1629	310	10/9*	2/1*	2/1*	2/1*	2/2*	4/2*	3/2*
**T30**	1343	2	0.15	0	0	2	1104	237	0/0*	0/0*	0/0*	0/0*	0/0*	0/0*	0/0*
**T32**	599	15	2.5	0	3	12	557	27	0/0*	2/0*	2/0*	1/0	1/0*	1/0*	2/0*
**T35**	499	2	0.40	0	0	2	465	32	0/0*	0/0*	0/0*	0/0*	0/0*	0/0*	0/0*
**T37**	1500	78	5.2	8	35	35	1060	362	9/9*	1/1*	1/1*	2/1*	2/1*	2/1*	1/0*
**T39**	1400	4	0.29	0	3	1	1257	139	0/0*	0/0*	0/0*	1/1*	0/0*	0/0*	1/1*
**T40.CA**	2180	354	16	90	141	123	1531	295	10/10*	1/1*	4/4*	2/2*	2/2*	4/4*	2/2*
**T40.CB**	2180	134	6.2	86	22	26	1751	295	0/0*	1/1*	1/1*	1/1*	1/1*	2/2*	1/1*
**T41**	1200	299	25	2	99	198	730	171	10/5*	4/1*	5/2*	3/1*	5/2*	5/1*	3/1*
**T46**	1699	24	1.4	0	0	24	1297	378	0/0*	1/0*	0/0*	0/0*	1/0*	1/0*	1/0*
**T47**	1051	600	57	278	301	21	388	63	10/10*	6/6*	8/8*	2/2*	6/6*	8/8*	3/3*
**T50**	1451	124	8.6	0	35	89	1141	184	0/0*	3/1*	2/0*	2/2*	1/1*	0/0*	1/1*

**T53**	1400	101	7.2	0	9	92	1090	209	3/0*	1/0*	1/0*	1/0*	3/0*	3/0*	3/0*
**T54**	1400	19	1.4	0	1	18	1195	185	0/0*	1/0*	0/0*	1/0*	0/0*	0/0*	0/0*

Table B. Comparison of the performance of CONSRANK, the contact-based (Clust-CONSRANK) and the ligand RMSD-based (RMSDClust-RMSD) clustering approaches on the SCORE_SET targets. The number of interfaces for which at least one acceptable/high-medium quality (*) solution has been selected by each scoring approach is reported.

Method	Total Interfaces with ≥ 1 NL/HM
**CONSRANK**	6/5*
**Clust-CONSRANK**	
$\mathbf{MC}^{200}$	11/7*
$\mathbf{MC}^{/5}$	9/6*
$\mathbf{MC}^{/10}$	11/8*
**RMSDClust-CONSRANK**	
$\mathbf{MC}^{200}$	10/7*
$\mathbf{MC}^{/5}$	9/6*
$\mathbf{MC}^{/10}$	11/8*

Table C. Scoring results for the analysed targets/interfaces. Columns 1-9: features of analysed targets: H, M, A, I and R indicate the high, medium-quality, acceptable, incorrect and removed models. NL stays for native-like, that is the sum of H, M and A models. Columns 10-16: results of the scoring with original CONSRANK and with a "redundancy removal" approach at different distance thresholds (from 25 to 80). In each column the total number of NL/H+M (*) models per target/interface is reported. Positive results are highlighted in cyan.

Target.Interface	# models	NL	%NL	H	M	A	I	R	CONSRANK	Redundancy removal					
										25	30	40	50	60	80
**T29**	2083	144	6.9	2	72	70	1629	310	10/9*	10/7*	10/5*	9/5*	8/3*	8/2*	2/1*
**T30**	1343	2	0.15	0	0	2	1104	237	0/0*	0/0*	0/0*	0/0*	0/0*	0/0*	0/0*
**T32**	599	15	2.5	0	3	12	557	27	0/0*	0/0*	0/0*	0/0*	0/0*	0/0*	0/0*
**T35**	499	2	0.40	0	0	2	465	32	0/0*	0/0*	0/0*	0/0*	0/0*	0/0*	0/0*
**T37***	1500	78	5.2	8	35	35	1060	362	9/9*	9/5*	7/3*	4/2*	4/2*	2/1*	2/1*
**T39**	1400	4	0.29	0	3	1	1257	139	0/0*	0/0*	0/0*	0/0*	0/0*	0/0*	0/0*
**T40.CA**	2180	354	16	90	141	123	1531	295	10/10*	10/9*	10/8*	10/7*	10/5*	10/3*	5/3
**T40.CB**	2180	134	6.2	86	22	26	1751	295	0/0*	0/0*	0/0*	0/0*	0/0*	0/0*	0/0*
**T41**	1200	299	25	2	99	198	730	171	10/5*	10/7*	10/5*	7/1*	6/0*	4/0*	3/0*
**T46**	1699	24	1.4	0	0	24	1297	378	0/0*	2/0*	1/0*	1/0*	2/0*	3/0*	2/0*
**T47**	1051	600	57	278	301	21	388	63	10/10*	10/10*	10/10*	7/6*	3/2*	3/1*	1/1*
**T50**	1451	124	8.6	0	35	89	1141	184	0/0*	1/1*	3/2*	4/2*	4/1*	5/1*	2/0*
**T53**	1400	101	7.2	0	9	92	1090	209	3/0*	5/0*	4/0*	5/0*	6/0*	3/0*	0/0*

**T54**	1400	19	1.4	0	1	18	1195	185	0/0*	0/0*	0/0*	0/0*	0/0*	0/0*	0/0*
**T72.1**	914	6	0.66	0	0	6	659	249	0/0*	0/0*	0/0*	0/0*	0/0*	0/0*	0/0*
**T79.1**	999	20	2.0	0	7	13	701	278	0/0*	0/0*	0/0*	0/0*	0/0*	0/0*	0/0*
**T79.2**	999	63	6.3	0	7	56	658	278	0/0*	0/0*	0/0*	0/0*	0/0*	0/0*	0/0*
**T79.3**	999	2	0.20	0	0	2	719	278	0/0*	0/0*	0/0*	0/0*	0/0*	0/0*	0/0*
**T86.1**	1010	30	3.0	0	5	25	942	38	0/0*	0/0*	0/0*	0/0*	0/0*	0/0*	1/0*
**T86.2**	1010	25	2.5	0	8	17	947	38	0/0*	0/0*	0/0*	0/0*	0/0*	0/0*	0/0*

Table D. Comparison between CONSRANK, Clust-CONSRANK and the Redundancy-removal approach: the umber of interfaces for which at least one acceptable/high-medium quality (*) solution has been selected by each scoring approach/threshold is reported.

Method	Total Interfaces with ≥ 1 NL/HM
**CONSRANK**	6/5*
**Clust-CONSRANK**	
**S^{25}**	10/4*
**S^{30}**	12/5*
**C^{40}**	10/7*

**C^{50}**	11/6*
**C^{60}**	12/7*
**C^{80}**	10/5*
**MC^{200}**	13/7*
**$MC^{/5}$**	11/6*
**$MC^{/10}$**	14/8*
**Redundancy-removal**	
**25**	8/6*
**30**	8/6*
**40**	8/6*
**50**	8/5*
**60**	8/5*
**80**	8/4*
